# Development of a Real-Time Polymerase Chain Reaction Assay to Detect *Mycoplasma pneumoniae* Using the Panther Fusion System

**DOI:** 10.3390/diagnostics16172788

**Published:** 2026-08-30

**Authors:** Jeeyong Kim, MinSup Lim, Eunji Lee, Woong Sik Jang

**Affiliations:** 1 Department of Laboratory Medicine, College of Medicine, Korea University, Seoul 02841, Republic of Korea; 2 Department of Health and Environmental Convergence Science, School of Health Science, Korea University, Seoul 02841, Republic of Korea; limminsup@naver.com; 3 BK21 Graduate Program, Department of Biomedical Sciences, College of Medicine, Korea University, Seoul 02841, Republic of Korea; lucy5303@korea.ac.kr; 4 Department of Laboratory Medicine, Korea University Guro Hospital, Seoul 08308, Republic of Korea; plasmid18@korea.ac.kr

**Keywords:** *Mycoplasma pneumoniae*, macrolide resistance, allele-specific real-time PCR, 23S rRNA mutation, Panther Fusion

## Abstract

**Background/Objectives**: *Mycoplasma pneumoniae* is a major cause of community-acquired pneumonia, and the increasing prevalence of macrolide-resistant *M. pneumoniae* (MRMP) has created a need for rapid assays that simultaneously detect the pathogen and resistance-associated mutations. This study developed and validated a fully automated laboratory-developed test (LDT) using the Hologic Panther Fusion^®^ system. **Methods**: The automated MP assay targeting domain V of the *23S rRNA gene* was implemented on the Panther Fusion^®^ platform. Analytical performance was evaluated by determining the limit of detection (LoD), repeatability, reproducibility, and cross-reactivity. Clinical performance was assessed using 419 nasopharyngeal swab specimens (90 positive and 329 negative) and compared with conventional real-time PCR. Mutation status was confirmed by sequencing. **Results**: The automated MP assay detected wild-type, A2063G, and A2064G targets with an LoD of approximately 1 × 10^2^ copies/μL and showed no cross-reactivity with 16 respiratory pathogens. Clinical sensitivity and specificity were 96.7% (87/90) and 100% (329/329), respectively, with high concordance with conventional real-time PCR and sequencing. **Conclusions**: The automated MP assay provides rapid and accurate detection of *M. pneumoniae* and major macrolide resistance mutations, offering a practical molecular diagnostic solution for routine clinical laboratories.

## 1. Introduction

*Mycoplasma pneumoniae* (MP) is a major pathogen responsible for community-acquired pneumonia (CAP) in children and adolescents, contributing significantly to the global burden of acute respiratory infections worldwide [1,2]. This pathogen exhibits a distinctive periodic epidemic pattern, typically resurging every 3 to 7 years, which triggers abrupt spikes in healthcare utilization, ranging from outpatient visits to emergency department admissions and prolonged hospitalizations [3,4]. Although a majority of *M. pneumoniae* infections follow a self-limiting clinical course, delayed or inaccurate diagnosis frequently leads to inappropriate empirical antibiotic use, protracted respiratory symptoms, and an elevated risk of severe extra-pulmonary complications. This underscores the paramount clinical necessity for rapid and highly accurate diagnostic modalities.

As *M. pneumoniae* (MP) intrinsically lacks a peptidoglycan cell wall, it is inherently resistant to beta-lactam antibiotics. Consequently, clinical management relies heavily on macrolides, tetracyclines, and fluoroquinolones, with macrolides historically serving as the primary first-line therapeutic regimen for pediatric cohorts [5]. However, the recent global dissemination of macrolide-resistant *M. pneumoniae* (MRMP) has emerged as a critical public health dilemma, with alarmingly high resistance rates concentrated in East Asian countries [6,7].

The epidemiological landscape of MRMP exhibits profound geographic heterogeneity. While resistance rates generally remain below 10% in Europe and North America, the Western Pacific and Asian regions report the highest prevalence of MRMP strains worldwide [6,8]. Mechanistically, this macrolide resistance is predominantly mediated by specific point mutations within domain V of the *23S rRNA gene*—most notably the A2063G and A2064G substitutions—which severely compromise drug binding and confer high-level resistance [9,10].

In Asia, a dramatic escalation in MRMP prevalence has been observed during major epidemic cycles. Outbreak data from China reveal resistance rates soaring above 70–90% [11,12], while recent epidemics in Japan demonstrate a resurgence of resistant clones, exceeding 50% in multiple surveillance studies [13,14,15]. Similarly, in the Republic of Korea, the prevalence of MRMP fluctuates in tandem with epidemic periods; recent outbreaks have been explicitly linked to blunted clinical responses to initial macrolide therapy, persistent fever, and extended hospital stays among pediatric and adolescent patients [16,17,18]. These dynamic shifts emphasize that timely, region- and period-specific resistance surveillance data are indispensable for optimizing localized empirical treatment algorithms.

Traditional laboratory diagnosis of *M. pneumoniae* infection has relied on serological assays, culture isolation, and nucleic acid amplification tests (NAATs). Serology is inherently limited in the acute phase due to the delayed kinetics of the antibody response and potential cross-reactivity, whereas culture is impractical for routine clinical decision-making due to the organism’s fastidious nature, low diagnostic sensitivity, and a protracted turnaround time (TAT) spanning several weeks [19,20]. Consequently, PCR-based NAATs have become the diagnostic gold standard owing to their superior sensitivity and specificity [21,22,23,24]. Furthermore, rapid genotypic identification of macrolide resistance at the time of initial diagnosis is imperative for guiding directed antimicrobial selection and reinforcing institutional antibiotic stewardship [25,26].

Recently, sample-to-result fully automated molecular platforms have revolutionized laboratory workflows by streamlining the entire process from raw specimen input to final result reporting [27]. The Open Access functionality of the Hologic Panther Fusion^®^ System (Hologic, Marlborough, MA, USA) permits clinical laboratories to seamlessly integrate laboratory-developed tests (LDT) into a high-throughput, fully automated paradigm. Utilizing this framework drastically reduces hands-on time to approximately 1.5 h, yielding a time to first result of under 4 h, even when multiplexing various in vitro diagnostic (IVD) assays and LDTs concurrently.

This study aimed to develop an automated MP test that detects *M. pneumoniae* and simultaneously confirms macrolide resistance by utilizing the Open Access capabilities of the Hologic Panther Fusion^®^ system. The analytical and clinical performance of the newly developed automated MP assay were evaluated and compared with those of a conventional real-time PCR assay. Additionally, an internal control analysis was included to assess sample integrity and the adequacy of the nucleic acid extraction and amplification processes. Furthermore, the usefulness of the newly developed automated MP assay in actual clinical laboratories was evaluated.

## 2. Materials and Methods

### 2.1. Study Design and Clinical Specimens

To evaluate the diagnostic performance of the newly developed automated MP assay, we conducted an analytical performance evaluation using plasmids. Clinical performance was evaluated by comparing the automated MP assay with a conventional real-time PCR assay, the Allplex PneumoBacter Assay (Seegene, Seoul, Republic of Korea), using a total of 419 clinical specimens. Furthermore, to detect common drug-resistant *M. pneumoniae* strains, a comparative evaluation between the automated MP assay and Sanger sequencing was also performed. This study was conducted using clinical specimens submitted to Korea University Ansan Hospital. A total of 419 nasopharyngeal swab specimens were collected from patients with respiratory infections between January 2023 and October 2025. Among these specimens, 90 were positive and 329 were negative for *M. pneumoniae*.

### 2.2. Primer and Probe Design for Mycoplasma pneumoniae

The complete sequences of the quantitative polymerase chain reaction (qPCR) primers and probes used for the detection of *M. pneumoniae* and the analysis of macrolide resistance are listed below (Table 1).

A2063G and A2064G were simultaneously detected using a multiplex probe-based real-time PCR assay targeting domain V of the *23S rRNA gene*.

Each probe was labeled with a distinct fluorescent reporter dye (FAM, HEX, Cy5, or Texas Red) and an appropriate quencher (BHQ1 or BHQ2), enabling multiplex signal detection within a single reaction.

The primer and probe set were implemented and optimized using the Open Access functionality of the Hologic Panther Fusion^®^ System. This platform integrates automated nucleic acid extraction with multi-channel real-time fluorescence detection, enabling detection of *M. pneumoniae* and macrolide resistance.

### 2.3. Automated MP Assay

An automated multiplex real-time PCR assay for the detection of *M. pneumoniae* and macrolide resistance-associated mutations was developed using the Open Access functionality of the Panther Fusion^®^ System (Hologic, Marlborough, MA, USA). The assay was designed to simultaneously detect *M. pneumoniae* and differentiate the wild-type (WT), A2063G, and A2064G sequences in the *23S rRNA gene*. The Panther Fusion System integrates automated nucleic acid extraction, PCR reagent preparation, amplification, and multi-channel fluorescence detection into a sample-to-result workflow.

For automated sample processing, 500 μL of each nasopharyngeal swab specimen was transferred into a Panther Fusion specimen lysis tube containing 710 μL of lysis buffer and loaded onto the system. A total of 360 μL of the resulting lysate mixture was subjected to nucleic acid extraction using Panther Fusion Extraction Reagent-S (Hologic). An internal control was included at the beginning of the extraction process to monitor nucleic acid extraction and amplification. The extracted nucleic acids were eluted in a final volume of 50 μL.

Optimization experiments were performed by systematically varying the concentrations of MgCl_2_ and the individual primers and probes. All buffer components were obtained from Hologic. Under the optimized reaction conditions, KCl and Tris (pH 8.0) were used at final concentrations of 50 and 8 mM, respectively. MgCl_2_ concentrations ranging from 1 to 5 mM were evaluated, and a final concentration of 3 mM was selected based on amplification efficiency, fluorescence signal reproducibility, and discrimination among the wild-type, A2063G, and A2064G targets.

The primers and probes listed in Table 1 were combined into a single working primer/probe mixture. The mixture contained the forward primer (Primer F) and reverse primer (Primer R) at 10 μM each, the wild-type and MP Pan probes at 5 μM each, the A2064G probe at 10 μM, and the A2063G probe at 12 μM. The concentrations of the individual primers and probes were adjusted to provide balanced amplification and fluorescence signals across the respective detection channels while minimizing non-specific amplification.

For the 12-test Open Access format, a 550 μL primer, probe, and buffer mixture was prepared by combining 427.97 μL of nuclease-free water, 44.69 μL of 1 M KCl, 3.10 μL of 1 M MgCl_2_, 5.50 μL of 1 M Tris (pH 8.0), and 68.75 μL of the working primer/probe mixture. The prepared mixture was loaded onto the Panther Fusion System and used with the Open Access RNA/DNA Enzyme Cartridge according to the manufacturer’s instructions.

During automated assay processing, the Panther Fusion System rehydrated the enzyme and nucleotide pellet with the prepared primer, probe, and buffer mixture and combined the reconstituted reagents with 5 μL of the extracted nucleic acid to produce a final reaction volume of 25 μL. Real-time PCR was performed with an initial denaturation at 95 °C for 1 min 59 s, followed by 40 cycles of denaturation at 95 °C for 9 s and annealing/extension at 65 °C for 40 s. Fluorescence signals were acquired during the annealing/extension step of each cycle.

**Figure 1:**
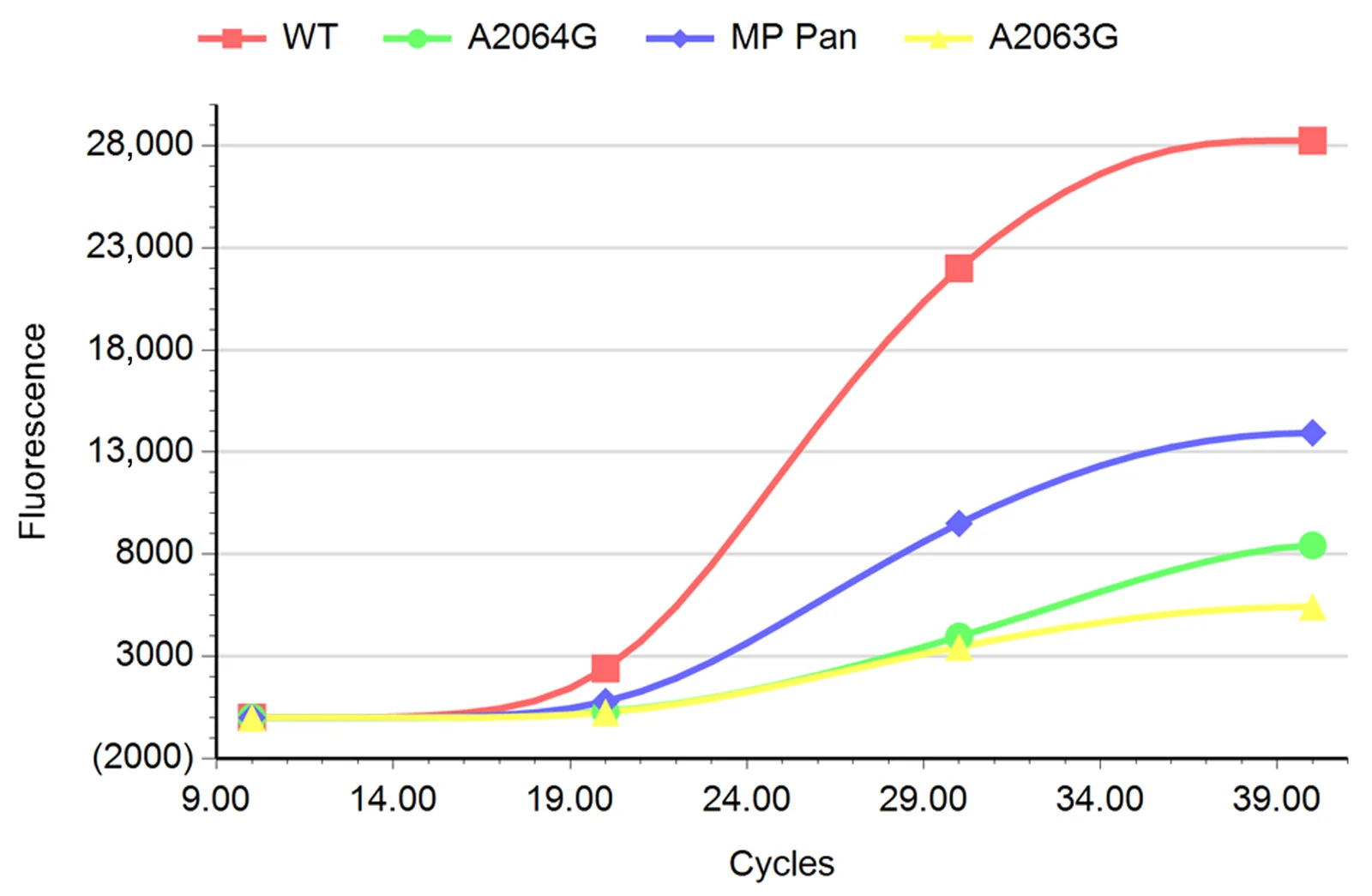
Threshold cycle of automated MP assay for *M. pneumoniae* detection.

The presence of *M. pneumoniae* and its 23S rRNA genotype (wild type, A2063G, or A2064G) was determined based on the corresponding fluorescence signals. The optimized conditions were used for subsequent analytical and clinical performance evaluations. (Figure 1).

### 2.4. Conventional Real-Time PCR Assay

To evaluate the clinical performance of the newly developed automated MP assay, the results were compared with those obtained using the Allplex™ PneumoBacter Assay, which is approved for clinical use in Korea. Nucleic acids were extracted from 200 μL of each nasopharyngeal swab specimen and eluted in 50 μL using the Genolution Nextractor^®^ automated nucleic acid extraction system (Genolution, Seoul, Republic of Korea). Five microliters of the extracted nucleic acid was added to the Allplex PneumoBacter Assay reaction mixture, and amplification was performed using the CFX96 Real-Time PCR Detection System (Bio-Rad, Hercules, CA, USA). Thermal cycling consisted of 35 cycles of 94 °C for 20 s, 58 °C for 20 s, and 72 °C for 20 s, followed by a final extension at 72 °C for 7 min. All procedures and result interpretations were performed according to the manufacturer’s instructions.

### 2.5. Sequencing

For Sanger sequencing, the *23S rRNA gene* region containing the macrolide resistance-associated mutation sites was amplified using the 23S rRNA forward and reverse primers listed in Table 1. PCR amplification was performed using the ELPIS 2× qPCR TaqMan Master Mix in a total reaction volume of 20 μL containing 5 μL of template DNA. Thermal cycling consisted of initial denaturation at 95 °C for 3 min, followed by 45 cycles of denaturation at 95 °C for 30 s and annealing/extension at 65 °C for 40 s. The resulting PCR products were subjected to Sanger sequencing using the corresponding primers. Sanger sequencing was used to confirm the 23S rRNA genotypes identified by the automated MP assay, including the wild-type, A2063G, and A2064G sequences.

### 2.6. Analytical Performance Evaluation

The analytical performance of the automated MP assay was evaluated to assess its ability to detect *M. pneumoniae* and macrolide resistance. The limit of detection (LoD), amplification efficiency, repeatability, and reproducibility were evaluated individually.

The LoD was determined using serial dilutions of quantified plasmid copies containing the wild-type, A2063 mutation, and A2064 mutation *M. pneumoniae* target sequences and was defined as the lowest concentration at which the target was consistently detected with a predefined positivity rate [28]. Amplification efficiency was evaluated to confirm stable PCR performance across the tested concentration range, and all targets demonstrated acceptable amplification characteristics under the established assay conditions. Repeatability was assessed by testing multiple replicates of the same samples within a single run, and consistent Ct-based classification of wild-type and macrolide-resistant targets was observed. Reproducibility was evaluated across different runs and days, demonstrating consistent differentiation between wild-type and A2063/A2064 mutant targets based on Ct values [29].

### 2.7. Clinical Performance Evaluation

Clinical performance was further assessed by calculating sensitivity, specificity and concordance rate using 419 clinical specimens, including 90 *M. pneumoniae* positive and 329 negative samples. The performance of the automated MP assay was compared with that of a conventional real-time PCR assay, demonstrating comparable diagnostic accuracy for the detection of *Mycoplasma pneumoniae*. For comparative analysis, a commercially available real-time PCR assay (Seegene, Seoul, Republic of Korea) was used according to the manufacturer’s instructions.

In addition, cross-reactivity was evaluated using *M. pneumoniae* negative specimens and specimens positive for other respiratory pathogens. The tested pathogens include *Bordetella pertussis*, *Candida albicans*, *Escherichia coli*, human parainfluenza virus types 1, 2, 3, and 4, human rhinovirus, *Klebsiella pneumoniae*, *Pseudomonas aeruginosa*, respiratory syncytial virus, *Staphylococcus aureus*, *Staphylococcus epidermidis*, *Streptococcus agalactiae*, *Streptococcus pneumoniae*, and *Streptococcus pyogenes*.

### 2.8. Statistical Analysis

The test results using the automated MP assay were evaluated in comparison with conventional real-time PCR results using the Allplex PneumoBacter assay, which is a clinically approved method for detecting *M. pneumoniae*. The diagnostic performance of the automated MP assay was evaluated for clinical sensitivity, specificity, positive predictive value (PPV), negative predictive value (NPV), and concordance rate. The concordance rate between the automated MP assay and Allplex PneumoBacter assay was calculated using inter-rater agreement statistics (kappa). A *p* value of <0.05 was considered statistically significant. All statistical analyses were performed using SPSS for Windows (version 22.0; IBM Corporation, NY, USA) and MedCalc version 22.016 (MedCalc software Ltd., Ostend, Belgium).

## 3. Results

Among a total of 419 nasopharyngeal swab specimens, 90 specimens were confirmed as MP-positive, while 329 specimens were MP-negative.

The analytical performance of the automated MP assay was evaluated using quantified plasmids containing wild-type (WT), A2063 mutation, and A2064 mutation *M. pneumoniae* target sequences. The automated MP assay successfully detected all three targets across the tested dilution ranges. At the concentration 1 × 10^2^ copies/μL, the targets were detected in 19 out of 20 replicates, meeting the 95% criterion for LoD determination in wild type, A2063G, A2064G, and Pan. The mean Ct values at the LoD level ranged from approximately 38 to 42 cycles, and correct target classification was consistently achieved. Amplification performance remained stable across the evaluated concentration range, and no assay failure or abnormal amplification patterns were observed (Table 2).

Repeatability testing demonstrated consistent Ct-based classification of wild-type and macrolide-resistant targets across replicate measurements within a single run. Reproducibility assessment across different runs and days showed stable differentiation between wild-type and A2063/A2064 mutant targets, indicating reliable assay performance under varying experimental conditions. In addition, the automated MP assay allows allele-specific discrimination between wild-type and A2063G mutant strains, representing a potential additional advantage of the assay.

Cross-reactivity was evaluated using *Mycoplasma pneumoniae*-negative specimens and specimens positive for other common respiratory pathogens. The tested panel included bacterial and viral respiratory pathogens, including *Bordetella pertussis*, *Candida albicans*, *Escherichia coli*, human parainfluenza virus types 1–4, human rhinovirus, *Klebsiella pneumoniae*, *Pseudomonas aeruginosa*, respiratory syncytial virus, *Staphylococcus aureus*, *Staphylococcus epidermidis*, *Streptococcus agalactiae*, *Streptococcus pneumoniae*, and *Streptococcus pyogenes*.

No cross-reactivity or non-specific amplification was observed with any of the tested non-target pathogens, demonstrating the high analytical specificity of the assay (Table 3).

The overall agreement between the automated MP assay and the Allplex PneumoBacter assay was 99.28%, with a Cohen’s kappa value of 0.98 (95% CI: 0.95–1.00). The clinical sensitivity of automated MP was 96.67% (90.57–99.31%), calculated using MedCalc version 22.016 software. According to sequencing results used for mutation confirmation, the genotype distribution among MP-positive specimens consisted of 5 wild-type (WT) cases and 85 cases harboring the A2063G mutation. Using the automated MP assay, all 5 WT specimens were correctly detected, and 82 of the 85 A2063G mutant specimens were concordant with the sequencing results. However, three specimens identified as A2063G-positive by sequencing were reported as negative by the automated MP assay, resulting in three discrepant cases. The clinical specificity of automated MP was 100.00% (98.87% to 100.00%). For the 329 MP-negative specimens, both the automated MP assay and Allplex PneumoBacter assay yielded negative results in all cases, and no false-positive reactions were observed. Taken together, both assays demonstrated high concordance with the sequencing results, with 100% agreement in the classification of MP-negative specimens. The positive predictive value was 100.00% (95.85% to 100.00%), and the negative predictive value was 99.09% (97.27% to 99.70%) (Table 4 and Table 5).

No systematic discrepancies were observed between the automated MP assay and the conventional real-time PCR assay (Seegene), indicating a high level of agreement for both pathogen detection and mutation-based classification in clinical specimens.

**Figure 2:**
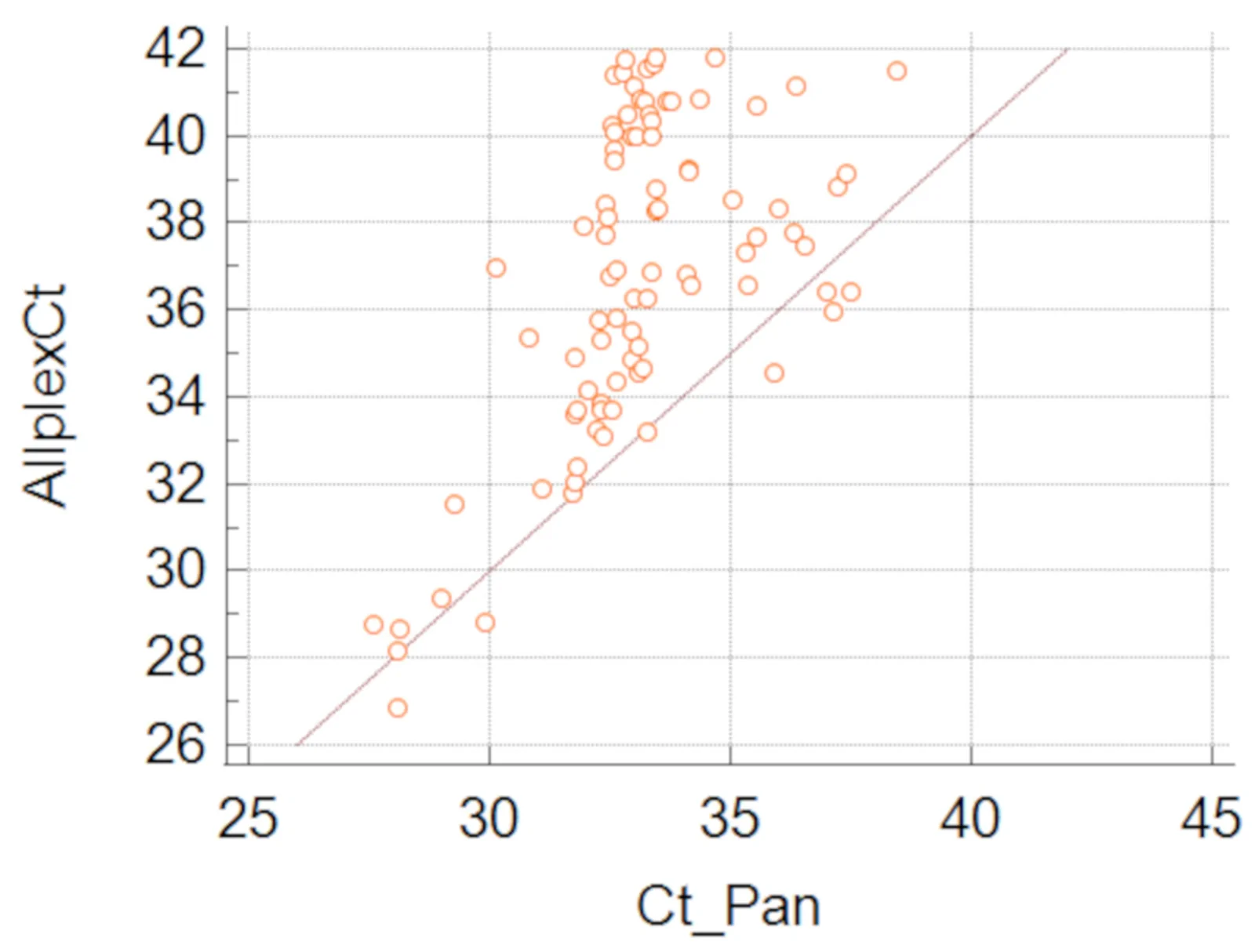
Correlation of threshold cycle of automated MP assay and Allplex PneumoBacter assay for *M. pneumoniae* detection.

For 90 positive specimens, the mean (± SD) cycle threshold (Ct) values of the automated MP assay and the Allplex PneumoBacter assay were 33.13 ± 2.12 and 36.82 ± 3.65, respectively. There was a moderate positive correlation in cycle threshold (Ct) values between the automated MP assay and the Allplex PneumoBacter assay (Pearson’s r = 0.60, *p* < 0.001) (Figure 2).

The detailed findings for the discordant cases are summarized in Table 6.

## 4. Discussion

The clinical management of *M*. *pneumoniae* infections has reached a critical turning point due to the relentless global spread of macrolide-resistant *M. pneumoniae* (MRMP) strains, particularly within East Asia [6,11]. In this clinical environment, conventional laboratory diagnostic methods, which have relied on demanding cultures or serological testing, are insufficient for acute treatment decisions [19]. Although nucleic acid amplification tests (NAATs) have established themselves as the diagnostic standard [21], there remains an urgent and unmet clinical need for high-throughput automated platforms capable of simultaneously identifying pathogens and analyzing antibiotic resistance profiles. This study is the first to validate the clinical validity of a real-time PCR-based laboratory-developed test (LDT) for the simultaneous detection of *M. pneumoniae* and screening for major 23S rRNA domain V mutations (A2063G and A2064G) in a fully automated sample-to-result workflow, utilizing the Open Access capabilities of the Hologic Panther Fusion^®^ system.

A rigorous assessment of analytical performance is a prerequisite for implementing any newly developed automated MP assay into routine laboratory operations. Our assay demonstrated robust analytical sensitivity and specificity across all target genotypes using quantified plasmid matrices. Notably, the limits of detection (LoDs) for the wild-type (WT), A2063G, and A2064G targets clustered consistently at the 1 × 10^2^ copies/μL threshold. At this near-single-copy boundary, we observed expected stochastic variations in amplification kinetics, particularly for the A2063G mutant plasmid, which yielded an 11/20 (55%) detection rate at 1 × 10^1^ copies/μL with an elevated mean Ct of 38.48 ± 2.79. Such minor discrepancies in amplification efficiency at extreme dilutions are well documented in multiplex assays and are typically caused by competitive probe binding or slight differences in thermodynamic stability between allele-specific primer/probe sets. Crucially, the assay maintained excellent linearity and reproducible Ct-based genotype segregation across higher concentration ranges 1 × 10^2^ copies/μL to 1 × 10^6^ copies/μL, with zero cross-reactivity or non-specific signal generation when challenged with a comprehensive panel of 16 common respiratory viral and bacterial pathogens (Table 5). This exceptional analytical specificity minimizes the risk of false-positive allocations, a critical attribute given the clinical overlapping of atypical pneumonia with other respiratory syndromes.

In clinical performance evaluation using 419 nasopharyngeal swab specimens, the automated MP assay demonstrated exemplary diagnostic concordance with both conventional real-time PCR and Sanger sequencing. The automated MP assay achieved a flawless 100% specificity, correctly categorizing all 329 non-infected specimens. In terms of clinical sensitivity, the assay successfully identified all 5 WT cases and 82 out of 85 sequencing-confirmed A2063G mutant cases, yielding an overall diagnostic sensitivity of 96.7% (87/90) for *M. pneumoniae* detection.

A meticulous analysis of the discrepancies in clinical cases is required. Three cases that tested positive by Sanger sequencing and qPCR but were not detected by the automated MP analysis were found to have very low bacterial levels, which was demonstrated by reference Ct values approaching or exceeding the detection limit (>38 cycles) of the manual analysis. Since the total volume of nucleic acid eluent input into the final master mix in high-throughput automated systems may differ slightly from manual settings, low-concentration clinical samples may be susceptible to sampling errors or stochastic non-amplification. However, it is clinically reassuring that no direct misclassification occurred among the detected strains; every single sample amplified by the automated MP assay was genotyped with 100% accuracy, matching the Sanger sequencing perfectly.

Furthermore, this assay demonstrates excellent allele-specific identification capabilities and clearly distinguished wild-type sequences from the A2063G mutation through individual target-specific fluorescence channels. These multiplexing capabilities possess significant clinical utility in identifying genomic heterogeneity within the host or co-infection with susceptible and resistant clones. This is a phenomenon often overlooked by conventional manual commercial assays, but it has a significant impact on fever reduction time and hospitalization duration in pediatric patients [17,30].

Beyond diagnostic accuracy, the successful migration of this automated MP assay onto the Hologic Panther Fusion^®^ platform offers transformative operational advantages that directly address the chronic bottlenecks of clinical laboratories. Traditional manual LDT workflows are notoriously fragmented, requiring batching of specimens to conserve reagents, extensive hands-on processing by skilled personnel, and separate instrumentation for extraction and amplification—all of which introduce significant windows for human error and cross-contamination [31]. By exploiting the Open Access architecture, we consolidated this custom assay into an interleaved, sample-to-result matrix. The system slashes hands-on time to roughly 1.5 h and accelerates the time to first result to under 4 h. Because the platform supports true random access and continuous sample loading, laboratories no longer need to accumulate a full batch of respiratory specimens before initiating a run [32,33]. This drastic reduction in turnaround time (TAT) is invaluable in urgent care and inpatient settings, where rapid, actionable data on macrolide resistance can avert the initial, inappropriate prescription of ineffective macrolides and prompt an immediate, targeted transition to second-line alternatives such as tetracyclines or fluoroquinolones [34].

Compared to the commercial Allplex™ PneumoBacter Assay, our novel automated MP assay demonstrates comparable diagnostic accuracy while providing key operational advancements. Specifically, it enables full automation of specimen pretreatment, which noticeably streamlines laboratory workflow and reduces total turnaround time (TAT). In addition to efficient detection of *M. pneumoniae*, our assay incorporates simultaneous identification of major macrolide resistance markers—a critical feature lacking in commercial diagnostic kits—making it a valuable tool for both rapid diagnosis and guided antimicrobial stewardship.

Several limitations of this study warrant consideration. First, because clinical validation was conducted solely on respiratory specimens (nasopharyngeal swabs/aspirates) archived at a single tertiary care center, the generalizability of the findings and epidemiological metrics across other geographical regions and alternative sample types—such as sputum or bronchoalveolar lavage fluid—remains to be fully established. Second, the small sample size of mutant strains (*n* = 85) constrained the statistical power needed to comprehensively assess diagnostic efficacy across diverse macrolide resistance genotypes. Although the assay accounts for the predominant A2063G and A2064G variants that represent the vast majority of worldwide resistance, rare domain V mutations (e.g., A2067N or C2611G) were insufficiently represented, highlighting the need for ongoing refinement as these minority variants emerge clinically. Finally, while no cross-reactivity with other species was detected during cross-reactivity evaluation, clinical specificity against Mycoplasma species such as *Mycoplasma genitalium* and *Mycoplasma hominis* could not be directly confirmed due to their absence in the negative clinical cohort. Consequently, multi-center prospective studies encompassing broader geographic regions, varied specimen types, and rare genotypes are essential to fully demonstrate the clinical utility of this diagnostic paradigm.

Consequently, this automated MP assay serves as a vital tool for institutional antimicrobial stewardship and epidemic preparedness. By linking real-time pathogen identification directly with its resistance genotype in an automated format, the clinical laboratory can provide clinicians with a definitive therapeutic roadmap within hours of admission. This preemptive diagnostic capability is poised to restrict the empirical, over-allocated use of broad-spectrum antibiotics, mitigate the selective pressure driving further MRMP evolution, and optimize healthcare resource allocation during seasonal respiratory surges [35,36]. A key advantage of fully automating this platform is the reduction in hands-on time and human error. In contrast to manual workflows, Panther Fusion^®^-based LDTs leverage end-to-end automation and built-in process checks to enhance result reliability, which is especially relevant for respiratory specimens with high variability [36,37,38].

## 5. Conclusions

In conclusion, we successfully developed and validated a fully automated laboratory-developed test (LDT) for the simultaneous detection of *M*. *pneumoniae* and the identification of the two major macrolide resistance-associated mutations (A2063G and A2064G) using the Open Access functionality of the Hologic Panther Fusion^®^ system. The automated MP assay demonstrated excellent analytical specificity, reliable analytical sensitivity, and high clinical concordance with conventional real-time PCR and sequencing-based genotyping. In addition, the automated MP assay accurately differentiated wild-type and macrolide-resistant strains within a single multiplex reaction while maintaining a fully automated sample-to-result workflow. This assay can be a practical and reliable molecular diagnostic platform for routine clinical laboratories. Its implementation has the potential to enhance diagnostic capacity, strengthen regional surveillance of macrolide-resistant *M. pneumoniae*, and contribute to more effective management of community-acquired respiratory infections.

## Figures and Tables

**Table 1:** Sequences of primers and probes used for the detection of *Mycoplasma pneumoniae* and the identification of macrolide resistance-associated mutations in the *23S rRNA gene*.

Target Gene	Name	Sequence (5′–3′)	μM
23S rRNA	F primer	CCAGGTACGGGTGAAGACAC	10
	R primer	AACAGCACACAACCAAGGGT	10
	Wild type Probe	[FAM]-CTTTCCGTCCCGTTGCGCC-[BHQ1]	5
	A2063G Mutant Probe	[Cy5]-CTTMCCGTCCCGTTGCGCC-[BHQ2] *	5
	A2064G Mutant Probe	[HEX]-CTCTCCGTCCCGTTGCGC-[BHQ1]	5
	Pan probe	[Texas Red]-GGACTTGTTGATGCGAAAGGTGGA-[BHQ1]	5

* Mixed Base: M represents A, C.

**Table 2:** Limit of detection (LoD) of the assay for wild-type and macrolide-resistant *M. pneumoniae*. The LoD was evaluated using serial dilutions of quantified plasmids containing wild-type (WT), A2063G mutation, A2064G mutation, and Pan for *M. pneumoniae*. Mean Ct values (± SD) are shown for each target. N/A indicates no amplification.

*M. pneumoniae* (Copies/μL)	Automated MP Assay			
	WT (Ct ± SD)	A2063G (Ct ± SD)	A2064G (Ct ± SD)	Pan (Ct ± SD)
1 × 10^6^	20.62 ± 0.72 (20/20)	20.04 ± 0.17 (20/20)	26.10 ± 0.28 (20/20)	20.74 ± 0.41 (60/60)
1 × 10^5^	24.16 ± 0.99 (20/20)	23.54 ± 0.13 (20/20)	29.56 ± 0.28 (20/20)	24.15 ± 0.41 (60/60)
1 × 10^4^	27.98 ± 0.46 (20/20)	27.69 ± 2.26 (19/20)	33.24 ± 0.30 (19/20)	28.78 ± 1.52 (60/60)
1 × 10^3^	31.46 ± 0.40 (20/20)	31.04 ± 0.23 (20/20)	36.99 ± 0.58 (20/20)	31.91 ± 0.30 (60/60)
1 × 10^2^	34.82 ± 0.92 (19/20)	34.67 ± 0.74 (20/20)	40.30 ± 1.48 (20/20)	35.78 ± 1.19 (59/60)
1 × 10^1^	38.16 ± 1.11 (15/20)	38.48 ± 2.79 (11/20)	41.94 ± 3.93 (7/20)	38.03 ± 1.22 (32/60)
1 × 10^0^	N/A	N/A	N/A	N/A
1 × 10^−1^	N/A	N/A	N/A	N/A
DW	N/A	N/A	N/A	N/A

**Table 3:** Cross-reactivity was tested against 16 non-*M. pneumoniae* respiratory pathogens. Ct values obtained from replicate measurements are shown. N/A indicates no amplification.

Pathogens	Automated MP Assay			
	WT	2063G	2064G	Pan
*Bordetella pertussis*	N/A	N/A	N/A	N/A
*Candida albicans*	N/A	N/A	N/A	N/A
*Escherichia coli*	N/A	N/A	N/A	N/A
Human parainfluenza virus type 1	N/A	N/A	N/A	N/A
Human parainfluenza virus type 2	N/A	N/A	N/A	N/A
Human parainfluenza virus type 3	N/A	N/A	N/A	N/A
Human parainfluenza virus type 4	N/A	N/A	N/A	N/A
Human rhinovirus	N/A	N/A	N/A	N/A
*Klebsiella pneumoniae*	N/A	N/A	N/A	N/A
*Pseudomonas aeruginosa*	N/A	N/A	N/A	N/A
Respiratory syncytial virus	N/A	N/A	N/A	N/A
*Staphylococcus aureus*	N/A	N/A	N/A	N/A
*Staphylococcus epidermidis*	N/A	N/A	N/A	N/A
*Streptococcus agalactiae*	N/A	N/A	N/A	N/A
*Streptococcus pneumoniae*	N/A	N/A	N/A	N/A
*Streptococcus pyogenes*	N/A	N/A	N/A	N/A

**Table 4:** Sensitivity and specificity of automated MP assay and Allplex PneumoBacter assay for *M. pneumoniae* detection.

Clinical Samples		Allplex PneumoBacter Assay	Automated MP Assay
*Mycoplasma* *pneumoniae*	Positive	90/90 (100%)	87/90 (96.67%)
	Negative	329/329 (100%)	329/329 (100%)

**Table 5:** Concordance of automated MP assay and sequencing for *Mycoplasma pneumoniae* detection.

Sequencing Genotype	Automated MP Assay				
	WT	A2063G	A2064G	Pan	Not Detected
*M. pneumoniae* wild type (*n* = 5)	5 (100%)	0	0	5 (100%)	0
*M. pneumoniae* A2063G (*n* = 85)	0	82 (96.47%)	0	82 (96.47%)	3 (3.53%)

**Table 6:** Discordant cases between Automated MP assay, Allplex PneumoBacter assay, and Sanger sequencing.

Sequencing	Automated MP					Allplex PneumoBacter	
Genotype	Result	Ct (Pan)	Ct (WT)	Ct (A2063G)	Ct (A2064G)	Result	Ct
A2063G	Negative	N/A	N/A	N/A	N/A	*M. pneumoniae*	41.14
A2063G	Negative	N/A	N/A	N/A	N/A	*M. pneumoniae*	39.19
A2063G	Negative	N/A	N/A	N/A	N/A	*M. pneumoniae*	41.57

N/A: No amplification.

## Data Availability

The raw data supporting the conclusions of this article will be made available by the authors on request.

## References

[1] Waites K.B., Talkington D.F. (2004). *Mycoplasma pneumoniae* and its role as a human pathogen. Clin. Microbiol. Rev..

[2] Wu Q., Pan X., Han D., Ma Z., Zhang H. (2024). New insights into the epidemiological characteristics of *Mycoplasma pneumoniae* infection before and after the COVID-19 pandemic. Microorganisms.

[3] Kim E.K., Youn Y.S., Rhim J.W., Shin M.S., Kang J.H., Lee K.Y. (2015). Epidemiological comparison of three *Mycoplasma pneumoniae* pneumonia epidemics in a single hospital over 10 years. Korean J. Pediatr..

[4] Upadhyay P., Singh V. (2024). *Mycoplasma pneumoniae* outbreak in 2023: Post-pandemic resurgence of an atypical bacterial pathogen. Cureus.

[5] Bradley J.S., Byington C.L., Shah S.S., Alverson B., Carter E.R., Harrison C., Kaplan S.L., Mace S.E., McCracken G.H., Moore M.R. (2011). The management of community-acquired pneumonia in infants and children older than 3 months of age: Clinical practice guidelines by the Pediatric Infectious Diseases Society and the Infectious Diseases Society of America. Clin. Infect. Dis..

[6] Darazam I.A., Rabiei M.M., Gharehbagh F.J., Hatami F., Shahrokhi S., Akhgarzad A., Nazhand H.A., Ebadi H., Zeininasab A.H., Kazeminia N. (2025). Recent macrolide resistance pattern of Mycoplasma pneumonia in the world: A systematic review and meta-analysis. Iran. J. Public Health.

[7] Xing F.-F., Chiu K.H.-Y., Deng C.-W., Ye H.-Y., Sun L.-L., Su Y.-X., Cai H.-J., Lo S.K.-F., Rong L., Chen J.-L. (2024). Post-COVID-19 pandemic rebound of macrolide-resistant *Mycoplasma pneumoniae* infection: A descriptive study. Antibiotics.

[8] Wang G., Wu P., Tang R., Zhang W. (2022). Global prevalence of resistance to macrolides in *Mycoplasma pneumoniae*: A systematic review and meta-analysis. J. Antimicrob. Chemother..

[9] Pereyre S., Goret J., Bébéar C. (2016). *Mycoplasma pneumoniae*: Current knowledge on macrolide resistance and treatment. Front. Microbiol..

[10] Wang N., Xu X., Xiao L., Liu Y. (2023). Novel mechanisms of macrolide resistance revealed by in vitro selection and genome analysis in *Mycoplasma pneumoniae*. Front. Cell. Infect. Microbiol..

[11] Chen Y., Jia X., Gao Y., Ren X., Du B., Zhao H., Feng Y., Xue G., Cui J., Gan L. (2024). Increased macrolide resistance rate of *Mycoplasma pneumoniae* correlated with epidemic in Beijing, China in 2023. Front. Microbiol..

[12] Li H., Li S., Yang H., Chen Z., Zhou Z. (2024). Resurgence of Mycoplasma pneumonia by macrolide-resistant epidemic clones in China. Lancet Microbe.

[13] Miyashita N., Ogata M., Fukuda N., Yamura A., Ito T. (2025). Macrolide-resistant *Mycoplasma pneumoniae* infection prevalence increases again in Osaka. Respir. Investig..

[14] Tanaka T., Oishi T., Miyata I., Wakabayashi S., Kono M., Ono S., Kato A., Fukuda Y., Saito A., Kondo E. (2017). Macrolide-resistant *Mycoplasma pneumoniae* infection, Japan, 2008–2015. Emerg. Infect. Dis..

[15] Oishi T., Kenri T., Yoshioka D. (2025). Macrolide-resistant *Mycoplasma pneumoniae* among Japanese children from 2008 to 2024. Microorganisms.

[16] Shin S., Koo S., Yang Y.-J., Lim H.-J. (2023). Characteristics of the *Mycoplasma pneumoniae* epidemic from 2019 to 2020 in Korea: Macrolide resistance and co-infection trends. Antibiotics.

[17] Lee J.K., Lee T., Kim Y.J., Kim D.R., Shin A., Kang H.M., Kim Y.J., Kim D.H., Eun B.W., Choe Y.J. (2024). Clinical manifestations, macrolide resistance, and treatment utilization trends of *Mycoplasma pneumoniae* pneumonia in children and adolescents in South Korea. Microorganisms.

[18] Lee E., Cho H.J., Hong S.J., Lee J., Sung H., Yu J. (2017). Prevalence and clinical manifestations of macrolide resistant *Mycoplasma pneumoniae* pneumonia in Korean children. Korean J. Pediatr..

[19] Loens K., Goossens H., Ieven M. (2010). Acute respiratory infection due to *Mycoplasma pneumoniae*: Current status of diagnostic methods. Eur. J. Clin. Microbiol. Infect. Dis..

[20] Gao L., Sun Y. (2024). Laboratory diagnosis and treatment of *Mycoplasma pneumoniae* infection in children: A review. Ann. Med..

[21] Loens K., Ieven M. (2016). *Mycoplasma pneumoniae*: Current knowledge on nucleic acid amplification techniques and serological diagnostics. Front. Microbiol..

[22] Nilsson A.C., Björkman P., Persson K. (2008). Polymerase chain reaction is superior to serology for the diagnosis of acute *Mycoplasma pneumoniae* infection and reveals a high rate of persistent infection. BMC Microbiol..

[23] Thurman K.A., Warner A.K., Cowart K.C., Benitez A.J., Winchell J.M. (2011). Detection of *Mycoplasma pneumoniae*, Chlamydia pneumoniae, and Legionella spp. in clinical specimens using a single-tube multiplex real-time PCR assay. Diagn. Microbiol. Infect. Dis..

[24] Zhou J., Xiao F., Fu J., Jia N., Huang X., Sun C., Xu Z., Zhang Y., Qu D., Wang Y. (2023). Rapid, ultrasensitive and highly specific diagnosis of *Mycoplasma pneumoniae* by a CRISPR-based detection platform. Front. Cell. Infect. Microbiol..

[25] Wolff B.J., Thacker W.L., Schwartz S.B., Winchell J.M. (2008). Detection of macrolide resistance in *Mycoplasma pneumoniae* by real-time PCR and high-resolution melt analysis. Antimicrob. Agents Chemother..

[26] Nummi M., Mannonen L., Puolakkainen M. (2015). Development of a multiplex real-time PCR assay for detection of *Mycoplasma pneumoniae*, Chlamydia pneumoniae and mutations associated with macrolide resistance in *Mycoplasma pneumoniae* from respiratory clinical specimens. SpringerPlus.

[27] Stellrecht K.A., Cimino J.L., Maceira V.P. (2020). The Panther Fusion system with open access functionality for laboratory-developed tests for influenza A virus subtyping. J. Clin. Microbiol..

[28] CLSI (2012). EP17-A2: Evaluation of Detection Capability for Clinical Laboratory Measurement Procedures.

[29] CLSI (2018). MM17: Validation and Verification of Multiplex Nucleic Acid Assays.

[30] Guiraud J., Ginevra C., Laurier-Nadalié C., Balcon C., Semanas Q., Gardette M., Jarraud S., Pereyre S., Bébéar C. (2026). Multiplex amplicon sequencing reveals polyclonality and low macrolide resistance in the French *Mycoplasma pneumoniae* outbreak, 2023–2024. Int. J. Infect. Dis..

[31] Kulis-Horn R.K., Tiemann C. (2020). Evaluation of a laboratory-developed test for simultaneous detection of norovirus and rotavirus by real-time RT-PCR on the Panther Fusion^®^ system. Eur. J. Clin. Microbiol. Infect. Dis..

[32] Hogan C.A., Sahoo M.K., Huang C., Garamani N., Stevens B., Zehnder J., Pinsky B.A. (2020). Comparison of the Panther Fusion and a laboratory-developed test targeting the envelope gene for detection of SARS-CoV-2. J. Clin. Virol..

[33] Zhen W., Smith E., Manji R., Schron D., Berry G.J. (2020). Clinical evaluation of three sample-to-answer platforms for detection of SARS-CoV-2. J. Clin. Microbiol..

[34] Murata M., Nishihara S., Kitagawa D., Onishi M., Mori T., Hachisuka S., Okubo T., Yamamoto N., Nishikawa H., Onaka M. (2025). Clinical impact of rapid test for macrolide-resistance gene mutation among cases with *Mycoplasma pneumoniae* in Japan. J. Infect. Public Health.

[35] Timbrook T.T., Morton J.B., McConeghy K.W., Caffrey A.R., Mylonakis E., LaPlante K.L. (2017). The effect of molecular rapid diagnostic testing on clinical outcomes in bloodstream infections: A systematic review and meta-analysis. Clin. Infect. Dis..

[36] Lucar J., Yee R. (2024). Diagnostic stewardship for multiplex respiratory testing: What we know and what needs to be done. Clin. Lab. Med..

[37] Mostafa H.H., Hardick J., Morehead E., Miller J.A., Gaydos C.A., Manabe Y.C. (2020). Comparison of the analytical sensitivity of seven commonly used commercial SARS-CoV-2 automated molecular assays. J. Clin. Virol..

[38] Craney A.R., Velu P.D., Satlin M.J., Fauntleroy K.A., Callan K., Robertson A., La Spina M., Lei B., Chen A., Alston T. (2020). Comparison of two high-throughput reverse transcription-PCR systems for the detection of severe acute respiratory syndrome coronavirus 2. J. Clin. Microbiol..

